# Risk of heart failure hospitalization among users of dipeptidyl peptidase-4 inhibitors compared to glucagon-like peptide-1 receptor agonists

**DOI:** 10.1186/s12933-018-0746-4

**Published:** 2018-07-17

**Authors:** Ghadeer K. Dawwas, Steven M. Smith, Haesuk Park

**Affiliations:** 1 Department of Pharmaceutical Outcomes and Policy, College of Pharmacy, University of Florida, PO Box 100495, Gainesville, FL 32610 USA; 2 Department of Pharmacotherapy and Translational Research, College of Pharmacy, University of Florida, PO Box 100486, Gainesville, FL 32610 USA; 3 Department of Community Health and Family Medicine, College of Medicine, University of Florida, PO Box 100237, Gainesville, FL 32610 USA

**Keywords:** Type 2 diabetes, DPP-4 inhibitors, GLP-1 agonists, Safety, Heart failure

## Abstract

**Background:**

Incretin-based therapies including dipeptidyl peptidase-4 (DPP-4) inhibitors and glucagon like peptide-1 (GLP-1) receptor agonists are novel medications for type 2 diabetes management. Several studies have found cardioprotective effects of incretin-based therapies; however, it remains unclear whether there is any difference in heart failure (HF) risk between the two incretin-based therapies (DPP-4 inhibitors and GLP-1 receptor agonists). We aimed to assess the risk of hospitalization due to HF with the use of DPP-4 inhibitors compared to GLP-1 receptor agonists.

**Methods:**

Using Truven Health Marketscan data, we conducted a retrospective cohort study of patients with type 2 diabetes, who were newly initiated on DPP-4 inhibitors or GLP-1 agonists. Follow-up continued from drug initiation until the first occurrence of: HF hospitalization (primary outcome), discontinuation of therapy (i.e. no fill for 7 days), switch to the comparator, end of enrollment, or end of study (December 2013). Cox proportional hazards models with propensity-score-matching were used to compare the risk of HF hospitalization between DPP-4 inhibitors and GLP-1 agonists.

**Results:**

A total of 321,606 propensity score-matched patients were included in the analysis (n = 160,803 for DPP-4 inhibitors; n = 160,803 for GLP-1 agonists). After adjusting for baseline characteristics and disease risk factors, the use of DPP-4 inhibitors was associated with a 14% decreased risk of HF hospitalization compared to GLP-1 agonists use [hazard ratio (HR), 0.86; 95% confidence interval (CI) 0.83, 0.90]. The results were consistent in patients without baseline HF (HR, 0.85; 95% CI 0.82, 0.89), but the association was not statistically significant for patients with baseline HF (HR, 0.90; 95% CI 0.74, 1.07).

**Conclusion:**

In this retrospective matched cohort of patients with type 2 diabetes, the use of DPP-4 inhibitors was associated with a reduced risk of HF hospitalization compared to GLP-1 agonists. However, the association was not statistically significant in patients who had HF prior to the use of DPP-4 inhibitors.

**Electronic supplementary material:**

The online version of this article (10.1186/s12933-018-0746-4) contains supplementary material, which is available to authorized users.

## Background

Dipeptidyl peptidase-4 (DPP-4) inhibitors, a relatively new class of diabetes medications, have been used widely since their approval in the United States in 2006 [1]. The class exerts antihyperglycemic effects by inhibiting the enzyme responsible for the degradation of glucagon-like peptide-1 (GLP-1) resulting in a reduction in both fasting and postprandial glucose concentration [2, 3]. Prior randomized clinical trials have proven these agents effective in reducing glycated hemoglobin (HbA1c), with a low risk of hypoglycemia and no clinically significant effect on weight [1, 4]. Despite the reported cardioprotective effect of DPP-4 inhibitors from pre-clinical trials, concerns emerged regarding their potential association with heart failure (HF) [1, 5]. For instance, a post hoc analysis from the Saxagliptin Assessment of Vascular Outcomes Recorded in Patients with Diabetes Mellitus–Thrombolysis in Myocardial Infarction 53 trial (SAVOR-TIMI 53) found a 27% increase in the risk of hospitalization due to HF with saxagliptin compared to placebo [6]. However, two other trials, the Examination of Cardiovascular Outcomes with Alogliptin Versus Standard of Care (EXAMINE) and Trial to Evaluate Cardiovascular Outcomes after Treatment with Sitagliptin (TECOS), reported no increased risk of hospitalization due to HF with alogliptin (EXAMINE) or sitagliptin (TECOS) compared to placebo [4, 7, 8]. Despite the uncertainty, the Food and Drug Administration (FDA) issued a warning of the increased risk of HF with two DPP-4 inhibitors: saxagliptin and alogliptin [9].

Subsequently, these trials were followed by several observational studies with conflicting results [1, 2, 4]. Fu et al. [10] found no significant difference in the risk of hospitalization due to HF between DPP-4 inhibitors and sulfonylureas in patients with baseline cardiovascular disease (CVD) (hazard ratio [HR], 0.95; 95% confidence interval (CI) 0.78, 1.15). Additionally, a more recent analysis found a protective effect for saxagliptin versus sulfonylureas (HR, 0.69; 95% CI 0.54, 0.87) and for sitagliptin versus sulfonylureas (HR, 0.61; 95% CI 0.50, 0.73) [2]. Given these equivocal findings and the limitations of prior studies such as the short follow-up time, there still a need for a better understanding of the risk of HF with DPP-4 inhibitors to guide prescribing decisions, especially in patients at elevated cardiovascular risk [2, 10]. Furthermore, given that sulfonylureas are not the only other second-line agent, important clinical questions remain, including whether differential risk of HF hospitalization exists between DPP-4 inhibitors and other preferred second line agents such as GLP-1 agonists. The latter have been recommended after metformin (± insulin in select patients) in recent guidelines, in part owing to an apparent all-cause mortality benefit observed in randomized trials [11, 12]. A recent meta-analysis of randomized clinical trials compared these two agents; however, the results were limited by the small number of events (9 total HF hospitalization events from 5 trials) [13]. Therefore, we sought to compare the risk of hospitalization due to HF of DPP-4 inhibitors compared with GLP-1 agonists in a large commercially-insured US population.

## Methods

### Data source

This was a retrospective cohort analysis using Truven Health Analytic MarketScan^®^ commercial and Medicare supplemental databases between January 2006 and December 2013. The databases include information on inpatient and outpatient medical claims, health expenditures, enrollment, and outpatient prescription claims of more than 150 million individuals. The commercial data includes privately insured employees and their dependents who are covered under plans including preferred provider organizations, health maintenance organizations, point-of-service plans, and indemnity plans. This study was approved by the University of Florida Institutional Review Board.

### Study population and exposure determination

New-users of DPP-4 inhibitors (sitagliptin, saxagliptin, alogliptin, or linagliptin) or GLP-1 agonists (exenatide, liraglutide, albiglutide, and dulaglutide) were identified based on the absence of any prior use during the baseline period (12 months lookback period). The start date of the first eligible prescription was used as the index date. To be included in the cohort, patients were required to have a diagnosis of type 2 diabetes mellitus [International Classification of Diseases, Ninth Revision, Clinical Modification (ICD-9-CM): 250.x0 or 250.x2] based on one inpatient or two outpatient encounters at two different service dates within 1 year prior to the index date, have 12 months of continuous enrollment in medical and pharmacy benefits prior to the index date, and be aged ≥ 18 years on cohort entry. Patients were excluded if they had a diagnosis of type 1 diabetes (ICD-9-CM: 250.x1 or 250.x3), end-stage renal disease (ESRD) (ICD-9-CM: 585.6), a prescription for DPP-4 inhibitors or GLP-1 agonists during the baseline period, or if they initiated both drugs on the same date. Further, to make our results comparable with those from the SAVOR-TIMI and other observational analyses, we excluded patients who had a hospitalization due to HF (ICD-9-CM: 402.×1, 404.×1, 404.×3, and 428.×) during the 60 days prior to the index date because a recent hospitalization due to HF is known to be highly predictive of subsequent HF exacerbations [2, 10].

Patients were considered exposed as long as they continued to refill their medication, allowing a gap no longer than 7 days between refills. Follow-up started from the index date until the first occurrence of one of the following censoring criteria: occurrence of the primary outcome (hospitalization due to HF), initiation of the study comparator (e.g. new users of DPP-4 inhibitors start or switch to GLP-1 agonists), discontinuation of therapy (i.e. no fill for 7 days), inpatient death (death was recorded if occurring during inpatient stay only), end of enrollment, or end of study period.

### Study outcome

The study outcome was the first hospitalization due to HF defined as an inpatient hospital claim with a primary or secondary diagnosis of HF (ICD-9-CM: 402.×1, 404.×1, 404.×3, and 428.××). This definition has been validated previously with a positive predictive value [PPV] > 90% [14].

We performed three secondary analyses, first, stratifying by the presence of prior diagnosis of HF defined as the occurrence of HF between 1 year and 60 days prior to the index date. Secondly, we stratified by the presence of prior CVD [cerebrovascular diseases, myocardial infarction (MI), stroke, coronary atherosclerosis, ischemic heart diseases, angina pectoris]. Thirdly, we assessed HF hospitalization risk of individual DPP-4 inhibitors, saxagliptin and sitagliptin, compared to GLP-1 agonists.

### Confounders adjustment

To adjust for confounders and disease risk factors, propensity score (1:1) matching to the nearest neighbor was used. To estimate the propensity score, we used a logistic regression model to obtain the predicted probability of receiving DPP-4 inhibitors versus GLP-1 agonists as a function of pre-specified baseline covariates. Included covariates were demographics (age, sex), presence of comorbidities (asthma, chronic obstructive pulmonary disease [COPD], chronic kidney disease [CKD], ischemic heart disease, depression, stroke, hypoglycemia, hyperlipidemia, cancer, hypertension, MI, HF), use of other diabetes medications (insulin, metformin, thiazolidinediones [TZDs], α-Glucosidase inhibitors, sodium glucose co-transporter-2 [SGLT2] inhibitors, meglitinides, sulfonylureas), use of other medications (angiotensin-converting enzyme [ACE] inhibitors, aldosterone receptor blockers, β-blockers, calcium channel blockers, loop diuretics, potassium sparing diuretics, thiazide diuretics, and direct vasodilators), and measures of healthcare utilizations (total number of inpatient visits, total number of outpatient visits). The presence of baseline comorbidities were defined based on the presence of ICD-9 codes in either inpatient or outpatient claims. These variables were drawn from a prior protocol that was developed by the Mini-Sentinel for the assessment of cardiovascular outcomes among patients with type 2 diabetes [15].

### Statistical analysis

Descriptive statistics were summarized using mean for continuous variables and proportion for categorical variables. Demographics and disease risk factors were compared between users of DPP-4 inhibitors and those of GLP-1 agonists using a Chi square test for categorical variables and independent *t* test for continuous variables. After propensity score matching, standardized differences were used to examine the balance in patient characteristics, where imbalance was defined as an absolute value higher than 0.2 [16, 17]. Proportional hazard models were used to obtain the HR and associated 95% CI after propensity score matching in the primary and secondary analyses. In secondary analyses, we examined the heterogeneity of treatment effect in patients with and without prior HF, with and without baseline CVD, and comparing saxagliptin and sitagliptin to GLP-1 agonists.

Sensitivity analyses were conducted to examine the robustness of the primary study results by changing the outcome definition to primary diagnosis only and, separately, extending the follow-up time to 45 days post-treatment discontinuation. Propensity score matching was performed for each of the secondary and sensitivity analyses. All analyses were performed using SAS 9.4 (SAS Institute Inc., Cary, NC).

## Results

### Patient characteristics prior matching

A flow diagram of the study population is included in the Additional file 1: Figure S1. Prior to matching, a total of 358,632 new-users of DPP-4 inhibitors, and 174,711 new-users of GLP-1 agonists were identified. Table 1 summarizes baseline characteristics comparing the new-users of DPP-4 inhibitors to GLP-1 agonists users. Prior to matching, there were significant differences in patient demographics and clinical characteristics.

**Table 1:** Demographics and clinical characteristics of new-users of DPP-4 inhibitors and GLP-1 agonists prior to propensity score matching

Patient characteristics before PS matching	DPP-4 inhibitors		GLP-1 agonists		*P-*value
	(n = 358,632)		(n = 174,711)		
Mean age, years (± SD)	59.0	(± 12.1)	52.9	(± 10.4)	< 0.001
Sex, n (%)					
Male	196,172	(54.7)	75,475	(43.2)	< 0.001
Female	162,460	(45.3)	99,236	(56.8)	
Type of DPP-4 inhibitors					
Saxagliptin	51,233	(14.3)	NA	–	–
Sitagliptin	286,259	(79.8)	NA	–	–
Alogliptin	20,720	(5.8)	NA	–	–
Linagliptin	409	(0.1)	NA	–	–
Comorbidities, n (%)					
Asthma	19,007	(5.3)	11,182	(6.4)	< 0.001
COPD	21,518	(6.0)	7163	(4.1)	< 0.001
CKD	24,028	(6.7)	4717	(2.7)	< 0.001
Ischemic heart disease	64,554	(18)	22,188	(12.7)	< 0.001
Depression	11,835	(3.3)	8386	(4.8)	< 0.001
Stroke	22,235	(6.2)	5591	(3.2)	< 0.001
Hypoglycemia	11,835	(3.3)	5765	(3.3)	0.320
Hyperlipidemia	196,889	(54.9)	95,567	(54.7)	0.354
Cancer	35,863	(10.0)	11,007	(6.3)	< 0.001
Hypertension	222,352	(62.0)	100,634	(57.6)	< 0.001
Acute myocardial infarction	5379	(1.5)	1223	(0.7)	< 0.001
Heart failure^a^	8966	(2.5)	1747	(1.0)	< 0.001
Prior cardiovascular diseases	81,051	(22.6)	27,255	(15.6)	< 0.001
Use of other diabetes medications, n (%)					
Metformin	210,876	(58.8)	108,845	(62.3)	< 0.001
TZDs	94,320	(26.3)	48,395	(27.7)	< 0.001
α-Glucosidase inhibitors	2152	(0.6)	1048	(0.6)	0.694
Insulin	35,863	(10.0)	39,485	(22.6)	< 0.001
Meglitinides	9683	(2.7)	4717	(2.7)	0.507
Use of other medications, n (%)					
ACE inhibitors	128,390	(35.8)	57,655	(33.0)	< 0.001
Aldosterone receptor antagonists	8966	(2.5)	4542	(2.6)	0.047
α-Blockers	24,746	(6.9)	7687	(4.4)	< 0.001
Angiotensin-receptor blockers	56,305	(15.7)	26,381	(15.1)	< 0.001
β-Blockers	104,721	(29.2)	40,358	(23.1)	< 0.001
Calcium-channel blockers	68,857	(19.2)	26,207	(15.0)	< 0.001
Loop diuretics	45,546	(12.7)	20,092	(11.5)	< 0.001
Potassium-sparing diuretics	9683	(2.7)	4892	(2.8)	0.233
Thiazide diuretics	6455	(1.8)	2795	(1.6)	< 0.001
Vasodilators	4304	(1.2)	1048	(0.6)	< 0.001
Healthcare utilization, mean (± SD)					
Mean number of outpatient visits	16.8	(± 15.9)	17	(± 14.6)	< 0.001
Mean number of inpatient visits	1.3	(± 0.7)	1.4	(± 0.9)	< 0.001

*ACE* angiotensin-converting enzyme, *CKD* chronic kidney disease, *COPD* chronic obstructive pulmonary disease, *DPP-4* dipeptidyl peptidase-4, *GLP-1* glucagon-like peptide-1, *PS* propensity score, *SD* standard deviation, *TZDs* thiazolidinediones

^a^ Excluding heart failure cases that occurred during the 60 day period prior to the index-date

### Patient characteristics after matching

After propensity score matching, a total of 321,606 patients were included in the analysis (n = 160,803 each for DPP-4 inhibitors and GLP-1 agonists users). Table 2 summarizes patient demographics and disease characteristics post-matching. In the propensity score matched cohort, patient demographics, including age (mean, 52.7 years vs. 53.0 years), proportion of men (44.7% vs. 44.1%), presence of comorbidities such as asthma (6.4% vs. 6.4%), hyperlipidemia (57.4% vs. 56.8%), and hypertension (59.6% vs. 59.5%), and prior use of medications including metformin (62.2% vs. 62.0%), TZDs (24.4% vs. 24.5%), and β-blockers (22.5% vs. 23.0%) were comparable between groups.

**Table 2:** Demographics and clinical characteristics in propensity-score matched cohorts of new-users of DPP-4 inhibitors and GLP-1 agonists

Patient characteristics after PS matching	DPP-4 inhibitors		GLP-1 agonists		Std-diff %^a^
	(n = 160,803)		(n = 160,803)		
Mean age, years (± SD)	52.7	(± 10.9)	53.0	(± 10.2)	− 0.01
Sex, n (%)					
Male	71,879	(44.7)	70,914	(44.1)	− 0.02
Female	88,924	(55.3)	89,889	(55.9)	
Comorbidities, n (%)					
Asthma	10,291	(6.4)	10,291	(6.4)	0.00
COPD	6432	(4.0)	6593	(4.1)	− 0.01
CKD	4342	(2.7)	4663	(2.9)	− 0.02
Ischemic heart disease	19,457	(12.1)	20,100	(12.5)	− 0.01
Depression	7397	(4.6)	7558	(4.7)	0.01
Hypoglycemia	4985	(3.1)	5146	(3.2)	0.01
Hyperlipidemia	92,301	(57.4)	91,336	(56.8)	0.00
Cancer	9809	(6.1)	10,291	(6.4)	− 0.01
Hypertension	95,839	(59.6)	95,678	(59.5)	0.00
Use of other diabetes medications, n (%)					
Metformin	100,019	(62.2)	99,698	(62.0)	0.01
TZD	39,236	(24.4)	39,397	(24.5)	0.00
α-Glucosidase inhibitors	804	(0.5)	804	(0.5)	0.00
Insulin	28,141	(17.5)	29,749	(18.5)	− 0.03
Meglitinides	3698	(2.3)	3698	(2.3)	0.00
Use of other medications, n (%)					
ACE inhibitors	53,226	(33.1)	53,065	(33.0)	0.00
Aldosterone receptor antagonists	3859	(2.4)	4020	(2.5)	− 0.01
α-Blockers	6915	(4.3)	7075	(4.4)	− 0.01
Angiotensin-receptor blockers	23,638	(14.7)	23,638	(14.7)	0.00
β-Blockers	36,181	(22.5)	36,985	(23.0)	− 0.01
Calcium-channel blockers	23,799	(14.8)	23,960	(14.9)	− 0.01
Loop diuretics	16,402	(10.2)	17,367	(10.8)	− 0.01
Potassium-sparing diuretics	4181	(2.6)	4181	(2.6)	− 0.01
Thiazide diuretics	2412	(1.5)	2412	(1.5)	0.00
Vasodilators	965	(0.6)	965	(0.6)	0.00
Healthcare utilization, mean (± SD)					
Mean number of inpatient visits	1.4	(± 0.9)	1.3	(± 0.7)	− 0.09
Mean number of outpatient visits	15.5	(± 14.5)	16.8	(± 14.5)	− 0.10

*ACE* angiotensin-converting enzyme, *CKD* chronic kidney disease, *COPD* chronic obstructive pulmonary disease, *DPP-4* dipeptidyl peptidase-4, *GLP-1* glucagon-like peptide-1, *PS* propensity score, *SD* standard deviation, *Std-diff* standardized difference, *TZDs* thiazolidinediones

^a^ Imbalance in standardized difference is defined as an absolute value > 0.20

### Incident heart failure hospitalization

**Fig. 1:**
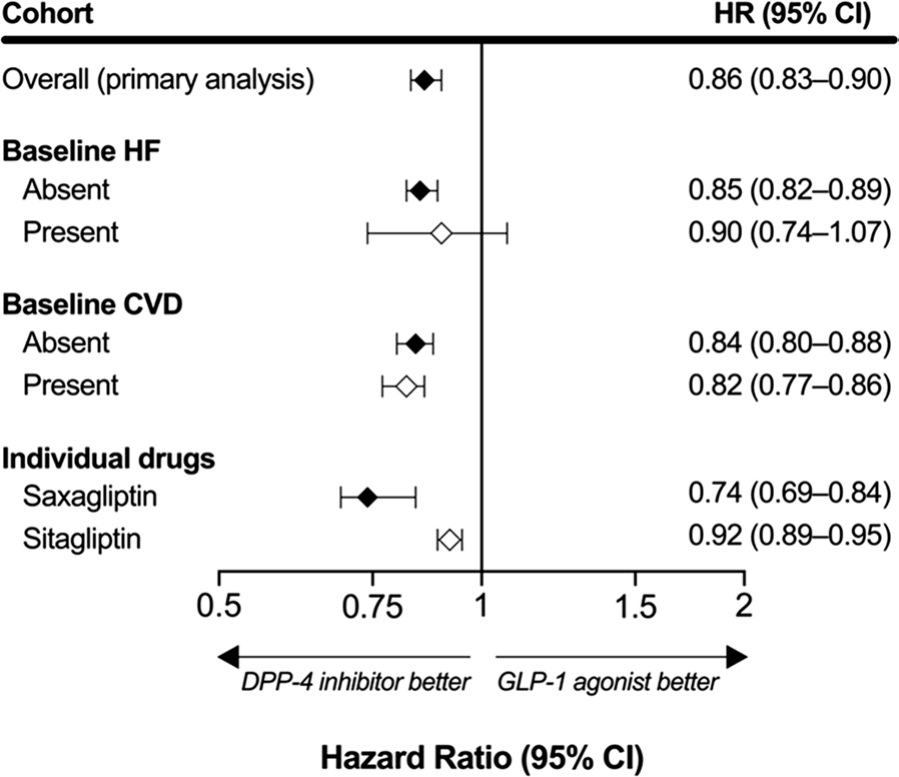
Risk of hospitalization due to heart failure with DPP-4 inhibitors versus GLP-1 agonists in propensity score matched analyses

The mean length of follow-up was 170 (± 290) days in the DPP-4 inhibitors group and 159 (± 285) days in the GLP-1 agonists group. Table 3 shows the unadjusted risk of hospitalization due to HF with DPP-4 inhibitors versus GLP-1 agonists users in the propensity score matched cohort. We identified 5714 of cases of HF hospitalization among DPP-4 inhibitors users (n = 160,803) and 6098 among GLP-1 agonists users (n = 160,803). The crude HF hospitalization incidence was 74 per 1000 person-years with DPP-4 inhibitors and 87 per 1000 person-years with GLP-1 agonists. In the proportional hazards model, use of DPP-4 inhibitors was associated with a 14% decreased risk of HF hospitalization compared to GLP-1 agonists use (HR, 0.86; 95% CI 0.83, 0.90), after adjusting for differences in baseline characteristics and disease risk factors (Fig. 1). Secondary analyses revealed similar results in patients without baseline HF (n = 158,543 for DPP-4 inhibitors; n = 158,543 for GLP-1 agonists; HR, 0.85; 95% CI 0.82, 0.89). However, among the comparatively small matched cohort of individuals with baseline HF (n = 1937 for DPP-4 inhibitors; n = 1937 for GLP-1 agonists), we observed no evidence of differential HF hospitalization risk (HR, 0.90; 95% CI 0.74, 1.07).

**Table 3:** Unadjusted risk of hospitalization due to heart failure with DPP-4 inhibitors versus GLP-1 agonists in propensity score matched analyses

Cohort	Medications	No. of patients	Person-years	No. of HF events	Crude incidence per 1000 person-years
Overall (primary analysis)	DPP-4 inhibitors	160,803	76,749	5714	74
	GLP-1 agonists	160,803	70,469	6098	87
Baseline HF					
Absent	DPP-4 inhibitors	158,543	75,545	5533	73
	GLP-1 agonists	158,543	69,682	5870	84
Present	DPP-4 inhibitors	1937	686	194	283
	GLP-1 agonists	1937	739	212	287
Baseline CVD					
Absent	DPP-4 inhibitors	132,047	62,552	3204	51
	GLP-1 agonists	132,047	58,366	3487	60
Present	DPP-4 inhibitors	28,085	13,544	2579	190
	GLP-1 agonists	28,085	11,835	2579	218
Individual drugs					
Saxagliptin	Saxagliptin	49,214	19,302	1335	69
	GLP-1 agonists	49,214	20,741	2058	99
Sitagliptin	Sitagliptin	160,609	81,573	6108	75
	GLP-1 agonists	160,609	70,441	6239	89

*CI* confidence interval, *CVD* cardiovascular diseases, *DPP-4* dipeptidyl peptidase-4, *GLP-1* glucagon-like peptide-1, *HF* heart failure, *HR* hazard ratio

### Sensitivity and subgroup analyses

Results of the unadjusted and adjusted secondary analyses are summarized in Table 3 and Fig. 1. We observed a 18% reduction in the risk of hospitalization due to HF among patients with baseline CVD (n = 28,085 in each exposure group) comparing DPP-4 inhibitors to GLP-1 agonists users (HR, 0.82; 95% CI 0.77, 0.86). Similar results were observed in those without baseline CVD (n = 132,047 in each exposure group).

We identified a total of 1335 cases of HF hospitalization among users of saxagliptin (n = 49,214) compared to 2058 cases among matched GLP-1 agonists users (n = 49,214); and 6108 cases among sitagliptin users (n = 160,609) compared to 6239 among GLP-1 agonists matched users (n = 160,609). The crude incidences of HF hospitalization were 69 per 1000 person-years in saxagliptin users and 99 per 1000 person-years in matched GLP-1 agonists users. For sitagliptin users, the crude incidences were 75 per 1000 person-years compared to 89 per 1000 person-years in the matched GLP-1 agonists users. In the proportional hazards models, we observed differential within-class effect comparing individual DPP-4 inhibitors to GLP-1 agonists. Saxagliptin, compared with GLP-1 agonists, reduced HF hospitalization by 26% (HR, 0.74; 95% CI 0.69, 0.84), whereas sitagliptin reduced HF hospitalization by 8% (HR, 0.92; 95% CI 0.89, 0.95) compared to GLP-1 agonists. Study results remained consistent in the sensitivity analyses (Table 4).

**Table 4:** Risk of heart failure with DPP-4 inhibitors versus GLP-1 agonists in propensity score matched sensitivity analyses

Study population	Medications	Person-years	No. of HF events	Crude incidence (per 1000 person-years)	Adjusted HR of HF (95% CI)	
Primary diagnosis only	DPP-4 inhibitors	77,070	3107	40	0.84 (0.80, 0.89)	
	GLP-1 agonists	70,706	3424	48	Reference	
Extend follow-up to 45 days	DPP-4 inhibitors	94,201	5714	61	0.89 (0.85, 0.92)	
	GLP-1 agonists	89,315	6098	68	Reference	

*CI* confidence interval, *DPP-4* dipeptidyl peptidase-4, *GLP-1* glucagon-like peptide-1, *HF* heart failure, *HR* hazard ratio

## Discussion

The evidence from observational data regarding the risk of hospitalization due to HF with DPP-4 inhibitors has been conflicting. For example, Fu et al. found no significant increase in the risk of hospitalization due to HF comparing DPP-4 inhibitors to sulfonylureas [1, 10], whereas other observational studies have reported lower risk with DPP-4 inhibitors compared to other antihyperglycemic drugs, including sulfonylureas and TZDs [2, 18, 19]. Our study builds on these prior observational studies and, to the best of our knowledge, is the first cohort study to compare the risk between the incretin mimetics. In this population-based matched cohort of patients with type 2 diabetes, we observed a 14% reduced risk of hospitalization due to HF among new-users of DPP-4 inhibitors, compared to new-users of GLP-1 agonists. The results were consistent in patients without baseline HF, but we were unable to confirm such an association in patients with baseline HF, in part because of a small sample size of patients with baseline HF (n = 3874).

A recent network meta-analysis compared the risk of HF hospitalization between DPP-4 inhibitors and GLP-1 agonists. Zheng et al. [13] assessed direct (pairwise comparisons) and indirect evidence (from DPP-4 inhibitors and GLP-1 agonists placebo-controlled trials). The authors reported that neither DPP-4 inhibitors, nor GLP-1 agonists, were associated with HF hospitalization when compared to placebo. However, they reported a reduced risk of HF hospitalization comparing GLP-1 agonists to DPP-4 inhibitors (HR, 0.82; 95% CI 0.70, 0.95) [13]. These results would appear to be directly contrary to our findings in the present study. But, further inspection of their results reveals interesting findings. First, their conclusion that GLP-1 agonists were associated with lower risks of HF hospitalization appear to have been driven largely by direct evidence which included only 9 total HF hospitalization events from 5 trials, resulting in a HR of 3.23 (comparing DPP-4 inhibitors to GLP-1 agonists) with very wide confidence intervals (0.70, 24.70). Conversely, indirect evidence from the placebo-controlled trials suggested the reverse: a protective effect of DPP-4 inhibitors compared to GLP-1 agonists. Furthermore, the authors reported significant inconsistency between the frequentist approach (results described above) and the Bayesian fixed-effect model, in which the latter revealed no difference in the risk of HF comparing DPP-4 inhibitors to GLP-1 agonists (HR, 1.21; 95% CI 0.91, 1.58).

### Possible mechanisms

The precise mechanism by which DPP-4 inhibitors may reduce HF exacerbation is not known. A recent single blinded randomized clinical trial found that DPP-4 inhibitors have no clinically meaningful effects on the B-type natriuretic peptide (BNP), a biomarker of HF. The study suggested that any changes in the level of HF-biomarkers over time is more likely to be due to the natural history of HF and not to be attributed to the effect of DPP-4 inhibitors [20]. However, there are several other possible mechanisms underlying the effect of DPP-4 inhibitors on HF. DPP-4 inhibitors are responsible for the degradation of several peptides that directly affect the heart and blood vessels [21]. One potential effect was observed in animal models where the continuous infusion of GIP led to the inhibition of the atherosclerotic lesion and the prevention of the penetration of macrophage in the aortic wall [21, 22]. The class also mediates control of the ratio of different receptor subtypes of neuropeptide Y (NPY) resulting in a regulation of blood pressure, blood flow, and inflammation [21]. Other studies suggest that DPP-4 inhibitors are associated with an improvement in left ventricular and endothelial functions leading to a delay in the development of HF, or worsening of HF, through its effect on blood pressure control, blood vessels, and cardiomyocytes [22–24].

### Clinical implications

Patients with type 2 diabetes are at higher risk of developing HF and the concomitant presence of both diseases can further complicate diabetes treatment. Thus, understanding the safety profile of diabetes medications—specifically the ones that were proven to be effective in reducing HbA1C level while maintaining body weight—is of particular interest to clinical practice. Our study found that despite documented safety warnings regarding the use of DPP-4 inhibitors in patients with HF, the class was not associated with an increased risk of HF compared with GLP-1 agonists. The findings complement the most recent findings from observational research and need to be considered within the context of other available alternative of diabetes therapy which have shown a lower risk of HF compared to DPP-4 inhibitors (i.e. SGLT2 inhibitors) [25]. Some may suggest that the observed protective effect of DPP-4 inhibitors on HF can be related to the higher risk of HF with the use of GLP-1 agonists. However, recent studies found no difference in the risk of HF when comparing GLP-1 agonists to placebo ruling out this alternative explanation [26–28].

### Strengths and limitations

The current study has several strengths. We used GLP-1 agonists as the comparator, and these agents represent reasonable second-line therapy for most patients according to existing guidelines. Secondly, we had a large sample size that allowed for the assessment of the heterogeneity of the treatment effect in selected subgroups of diabetes patients. Third, we performed several subgroup and sensitivity analyses providing confidence in the robustness of our primary findings. Nevertheless, the current study needs to be viewed in light of several limitations. First, the current study was conducted using observational data and, because of different route of administration (DPP-4 inhibitors are administered orally whereas GLP-1 agonists are injectable) as well as slightly different A1c lowering ability and adverse drug effects (e.g. weight loss) between DPP-4 inhibitors and GLP-1 agonists, the potential of selection bias may exist. However, we used a propensity score matching approach to minimize differences in baseline characteristics and disease risk factors between the two treatment groups. Second, deaths occurring outside of hospitals were not available in our data. Recent data suggest that GLP-1 agonists may have a mortality benefit compared with DPP-4 inhibitors, although the results of the analysis were limited by the small number of events (n = 20 in 7 trials) [13]. This, if true, could have led to survivor bias in our analysis, whereby GLP-1 agonists users may be less likely to die prior to a HF hospitalization, leading to more cases in this group. Nevertheless, we suspect any such bias to be negligible because the censoring rate due to end of enrollment (which include patients who died) was similar between the two groups (4% for DPP-4 inhibitors vs. 4% for GLP-1 agonists).

Third, the claims data used in this study were collected primarily for billing purposes and do not include clinical biomarkers, such as HbA1c. Thus, residual confounding resulting from missing data on important confounders such as demographics (e.g. race), or lab values (e.g. HbA1c) is still possible. Fourth, because outcome definition and the identification of confounders relies on ICD-9 codes only, there is the potential for bias resulting from errors in coding. However, the validity of ICD-9 codes used for the identification of the study outcome or the confounders has been assessed previously. Fifth, although informative censoring resulting from treatment discontinuation can impact the study results, our sensitivity analyses following the patients for a fixed period of time (45 days) provides some degree of confidence that this was not a major issue in our primary findings. Results from the current study are generalizable to patients with type 2 diabetes who are covered by commercial or Medicare supplemental insurance, limiting the generalizability to the broader Medicare population.

## Conclusion

In this retrospective matched cohort of patients with type 2 diabetes, the use of DPP-4 inhibitors was associated with a reduced risk of HF hospitalization compared to GLP-1 agonists, although the association was not statistically significant in patients with baseline HF. Furthermore, we found consistent beneficial effects comparing individual DPP-4 inhibitors including saxagliptin and sitagliptin to GLP-1 agonists.

## Additional file

**Additional file 1: Figure S1.** Flow diagram of the study population.

## Abbreviations

CVD: cardiovascular diseases
CI: confidence interval
DPP-4: dipeptidyl peptidase-4
ESRD: end-stage renal disease
FDA: Food and Drug Administration
GLP-1: glucagon-like peptide-1
HR: hazard ratio
HF: heart failure
ICD-9-CM: International Classification of Diseases, Ninth Revision, Clinical Modification
TZA: thiazolidinediones
