# Supplementary figures and images for: Risk of heart failure hospitalization among users of dipeptidyl peptidase-4 inhibitors compared to glucagon-like peptide-1 receptor agonists
Source: Cardiovasc Diabetol. 2018 Jul 17;17:102. doi: 10.1186/s12933-018-0746-4 (PMC6048850; doi:10.1186/s12933-018-0746-4)

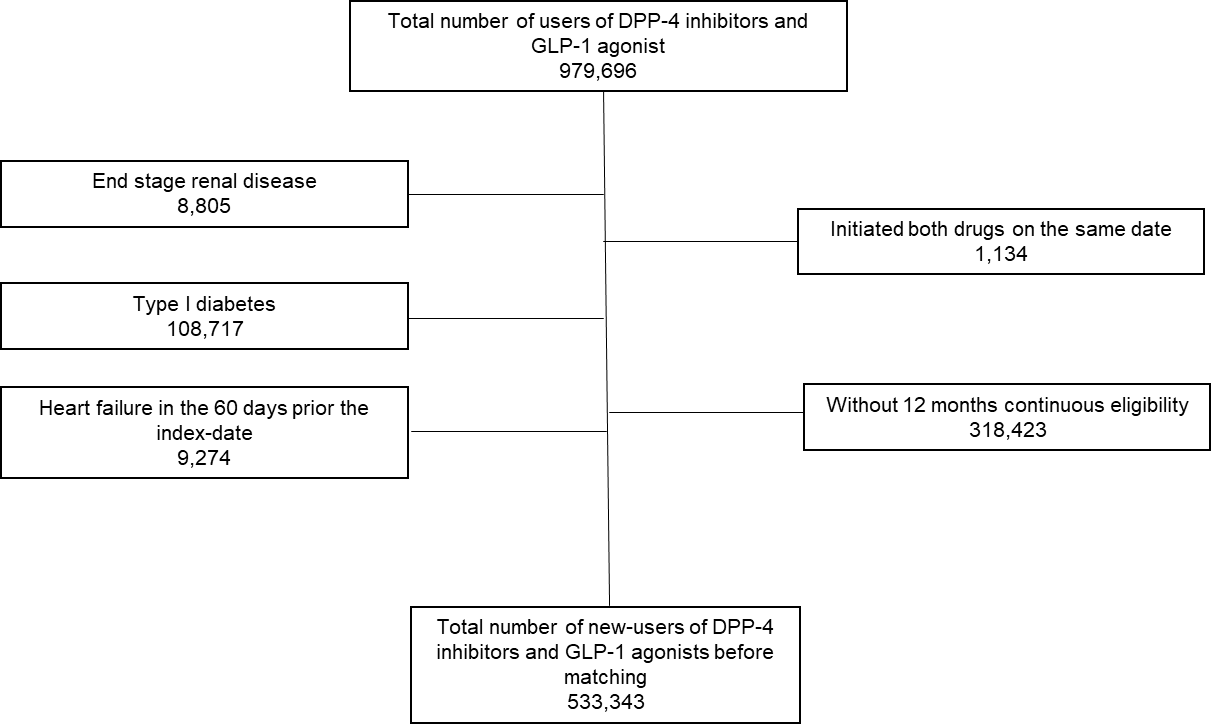


Additional file 1: Figure S1. Flow diagram of the study population

Supplement: Supplementary file 1 — Additional file 1: Figure S1. Flow diagram of the study population. [file 12933_2018_746_MOESM1_ESM.docx]
